# Phyto-therapeutics as anti-cancer agents in breast cancer: Pathway targeting and mechanistic elucidation

**DOI:** 10.1016/j.sjbs.2024.103935

**Published:** 2024-01-20

**Authors:** Abdullah Almilaibary

**Affiliations:** Department of Family and Community Medicine, Faculty of Medicine, Albaha University, Albaha, Saudi Arabia

**Keywords:** Breast cancer, TNBC, Signaling pathways, Phytochemicals, Anticancer agents, Medicinal plants

## Abstract

Cancer of the breast is the mainly prevalent class of cancer in females diagnosed over the globe. It also happens to be the 2nd most prevalent reason of cancer-related deaths among females worldwide. Some of the most common type’s therapies for carcinoma of the breast involve radiation therapy, chemotherapy, and resection. Many studies are being conducted to develop new therapeutic strategies for better diagnosis of breast cancer. An enormous number of anticancer medications have been developed as a result of growing understanding of the molecular pathways behind the advancement of cancer. Over the past few decades, the general survival rate has not greatly increased due to the usage of chemically manufactured medications. Therefore, in order to increase the effectiveness of current cancer treatments, new tactics and cutting-edge chemoprevention drugs are required. Phytochemicals, which are naturally occurring molecules derived from plants, are important sources for both cancer therapy and innovative medication development. These phytochemicals frequently work by controlling molecular pathways linked to the development and spread of cancer. Increasing antioxidant status, inactivating carcinogens, preventing proliferation, causing cell cycle arrest and apoptosis, and immune system control are some of the specific ways. This primary objective of this review is to provide an overview of the active ingredients found in natural goods, including information on their pharmacologic action, molecular targets, and current state of knowledge. We have given a thorough description of a number of natural substances that specifically target the pathways linked to breast carcinoma in this study. We've conducted a great deal of study on a few natural compounds that may help us identify novel targets for the detection of breast carcinoma.

## Highlights

• Present research is focused on creating more individualized and accurate therapy solutions to address the issues that occur in conventional treatment approaches for breast cancer.
• One of the important category of compounds that are best for breast cancer treatment include phytotherapeutic compounds.
• Several molecular targets and signalling pathways connected to the instigation and succession of cancer can be targeted by natural substances.
• This review focuses on the description of the molecular function played by a number of significant natural chemicals in breast cancer.

## Graphical abstract

**Figure:**
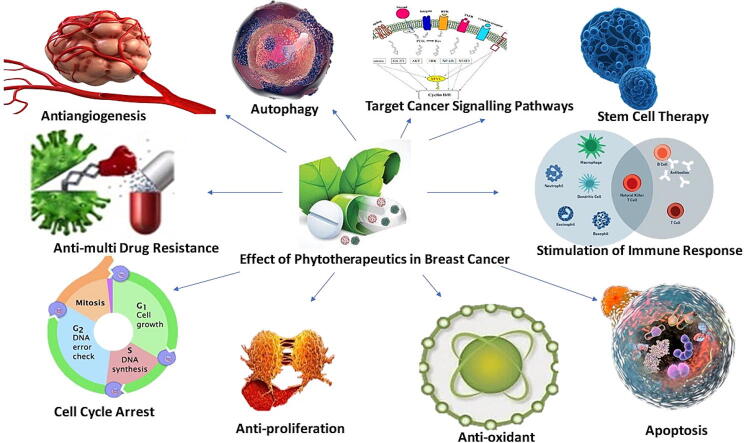

## 1 Introduction

Breast carcinoma is most typically detected in women globally (Li et al., 2017, Iqbal and Sharif, 2023). Furthermore, it is the 2nd leading cause of cancer-related mortality worldwide for females (Kamaruzman et al., 2018). In India, it is the cause of one-fifth of all cancers among the females and seven percent of the world's breast cancer cases. By 2030, epidemiological research indicates that there will be over two million cases of BC worldwide (Mehraj et al., 2023). When diagnosing breast carcinoma, the most often used treatments include radiation, chemotherapy, surgery, and hormone therapy. Drugs used in chemotherapy are often used to diagnose breast carcinoma, but their effectiveness is restricted by the emergence of multi-drug resistance, the disease's recurrence, and the frequency of side effects (Chen and Li, 2015, Ko and Moon, 2015, Singh et al., 2017, Kamaruzman et al., 2018). Drug resistance to multiple medications (MDR) is one of the most severe issues with the current diagnostic strategy (Doyle et al., 1998, Ullah, 2008, Mehraj et al., 2021b). The survival rate of those with breast carcinoma is quite low as a result of this. Numerous studies are currently being conducted to specifically develop an alternative breast cancer treatment system that can be utilized as novel therapeutic regime (Adler and Fosket, 1999, Burstein et al., 1999, Henderson and Donatelle, 2004, Boon et al., 2007, Mehraj et al., 2022a, Mehraj et al., 2022b). The many routes or processes that are frequently implicated in the development of breast carcinoma and its progression must be taken into consideration while developing new regimens (MacMahon et al., 1973, Miller and Bulbrook, 1980). A substance produced from a natural source that can target any of these pathways may be developed as a therapeutic strategy (Shoeb, 2006). Nearly, seventy-five percent of anticancer medications developed to date are derived from plants, which make up more than half of all drugs created to date (Newman and Cragg, 2016). Given the rising number of cancer-related fatalities, developing effective treatment options with zero resistance and minimal cytotoxic side effects is imperative. Additionally, using the natural cancer treatments like phytochemicals has demonstrated encouraging outcomes in the diagnosis of a range of malignant tumors (Shanmugam et al., Nobili et al., 2009, Mehraj et al., 2022b). Due to their great selectivity, low outlay, and low toxicity natural compounds and their derivatives therefore have the potential to be better chemotherapeutic agents (Fv Torquato et al., 2017). Despite extensive understanding of the molecular mechanisms behind the cancer-associated cellular signaling pathways, there are few chemotherapeutic strategies that specifically target oncogenic biomarkers (Sanchez-Vega et al., 2018, Mir et al., 2020). Therefore, to find possible natural compounds that specifically target the aberrantly activated proteins in breast cancer is important. Since numerous natural substances have demonstrated a variety of anticancer properties in various cancer types (Mir and Agrewala, 2008).

Different forms of tumours have altered cell signalling pathways. Cellular signalling pathways are intricate webs of interacting molecules that communicate with one another to control the biological functions of individual cells. Different growth factor receptors provide information to cells, which they then integrate to control various cellular functions as motility, differentiation, polarity, proliferation, apoptosis, and protein synthesis (El-Tanani et al., 2023). Different cell types undergo distinct modifications that are governed by signalling pathways, which also affect cellular development. Numerous signalling pathways control the motility, proliferation, and survival of cells. Thus, when an inhibitor blocks a mutationally triggered route, cancer cells can spread via a different signalling pathway (Bahar et al., 2023). Therefore, effective combination therapies, such as signalling inhibitors or a combination of signalling inhibitors with chemotherapeutic drugs that damage DNA, are necessary for an advanced cancer treatment. Consequently, comprehending the complexity of these pathways is crucial for researching the behavior of tumour cells. Numerous pathways, including the PI3K/Akt, Ras/MAP-Kinase, and cell cycle pathways, have been found to be often changed in cancer (Sofi et al., 2023). Furthermore, variations in the Wnt/β-catenin signalling have been noted in a variety of tumour forms. Even though the molecular mechanisms underlying these cancer-associated cellular signalling pathways are well understood, there are few chemotherapeutic strategies that specifically target oncogenic biomarkers (Hashem et al., 2022, Sofi et al., 2022a). Thus, to find possible treatment targets, a detailed grasp of the alterations in cell signalling pathways is necessary. Numerous natural substances have demonstrated a wide range of anticancer properties in various cancer types (Mehraj et al., 2022a, Mehraj et al., 2022b, Jan et al., 2023). These substances work against multiple signalling pathways, primarily those involved in cell death (apoptosis and autophagy) and embryonic development (Notch pathway, Wnt pathway, and Hedgehog pathway) (Wu et al., 2023). The potential of natural compounds to be promising novel products for cancer treatment makes them an important area of research. One of the best ways to combat cancer, including breast cancer, among the various therapeutic options currently accessible, is to use chemical formulations or chemicals derived from natural sources. Currently on the market, almost 60 % of cancer medications come from natural sources (Alharbi et al., 2022). Phytochemicals have few side effects and a high cancer effectiveness. Medications produced from plants can function as antioxidants, mitotic disruptors, histone deacetylase inhibitors, methyltransferase inhibitors, or drugs that prevent DNA damage (Rampogu et al., 2022). This review throws light upon the importance of natural products (phytotherapeutics) in blocking the several signalling pathways that act as catalysts for breast cancer development thereby opening the door for the creation and discovery of anticancer medications. Also we have presented a thorough description of the molecular function played by a number of significant natural chemicals in breast cancer in this study.

## 2 Phytochemicals targeting various pathways in breast cancer

**Fig. 1:**
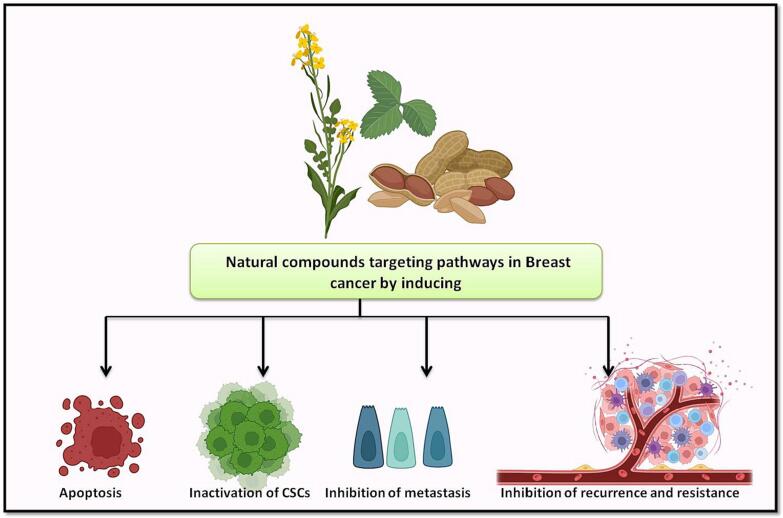
The natural compounds in breast cancer prevention. The use of natural compounds as anti-cancer therapeutics against breast cancer, the natural compounds target the aberrant activated pathways and proteins in breast cancer and induce apoptosis, inhibit the epithelial-mesenchymal transition and activation of cancer stem cells and also reduce the chemo-resistance and relapse of the disease.

The relationship between phytochemicals, particularly phenolics, and their potential to prevent cancer is a complex and widely researched topic. Plants generate bioactive substances called phytochemicals, which are frequently linked to a number of health advantages, including possible anticancer effects. Because of their antioxidant qualities, phenolics have attracted a lot of interest among these phytochemicals. Reactive oxygen species, another name for free radicals, are substances that can damage cells and DNA. Antioxidants are compounds that can neutralise these molecules. The hypothesis behind phenolics' putative anticancer properties is intimately related to their antioxidant capacities. Phenolics may shield cells from oxidative stress, an event associated with the onset of more than a few diseases, including cancer (Chirumbolo et al., 2018). It has been discovered that phytochemicals—bioactive substances obtained from plants—activate the Nrf2-ARE pathway. When they accomplish this, they can raise the production and action of antioxidant enzymes such as CAT, GPX, and SOD. As it strengthens the cellular antioxidant defence system and aids in shielding cells from oxidative damage—a process connected to the start and advancement of several illnesses, including cancer—this activation is seen as advantageous in the context of chemoprevention. One theory regarding phytochemicals' possible chemopreventive impact is that they can trigger the Nrf2-ARE pathway and modify the manufacture of antioxidant enzymes (Yang et al., 2014, Cook, 2018, Qiu et al., 2018). A vast array of intracellular targets is targeted by phytochemicals, and the way in which they interact with many of these targets remains a mystery to this day. Plant-derived phytoestrogens, including catechins, can activate survival signalling systems that are engaged during mitohormesis, in C. elegans, therefore inducing mitochondrial biogenesis and restoring mitochondrial function. By focusing on the mitochondria, these compounds should also be able to show that they can regulate cell morphogenesis and enhance mitochondrial biogenesis and function (Steib et al., 2014, Xiong et al., 2018). Phytochemicals are thought to affect metastasis by arresting cell cycle, inducing apoptosis, and reactivating tumor-suppressing genes by concentrating on critical kinases, transcription factors, and growth factor receptor driven pathways Fig. 1 (Landis-Piwowar et al., 2008, Aggarwal et al., 2015, Chen and Li, 2015). Natural substances have a lot of promise for breast cancer chemoprevention compared to synthetic medications because they show fewer adverse effects and low toxicity in several studies (Ranjan et al., 2019, Mehraj et al., 2021a, Mir et al., 2021). The following sections emphasize the value of several natural substances in preventing breast cancer in detail by targeting abnormal cell signaling pathways.

## 3 Genistein

**Fig. 2:**
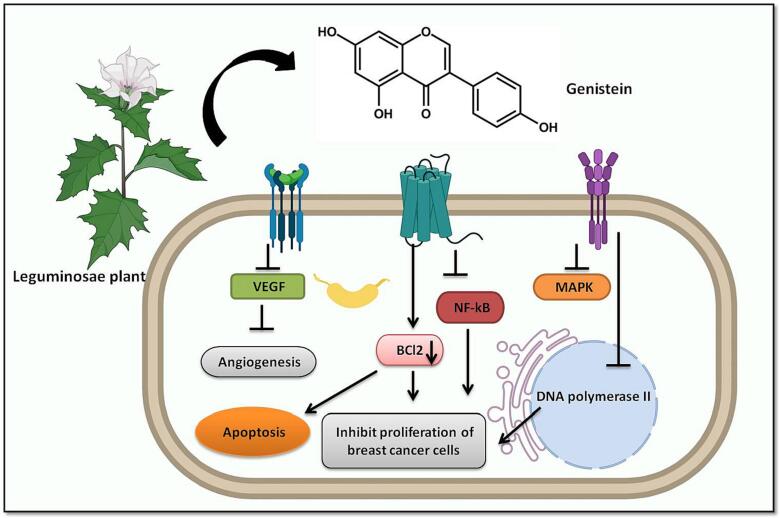
Schematic representation of natural compound Genistein targeting breast cancer signaling pathways. The natural compound Genistein prevents angiogenesis via altering the VEGF, NF-kB, and MAPK genes. Genistein also is known to induce apoptosis by inhibiting Bcl-2 and several other pro-apoptotic proteins. It also promotes cell cycle arrest by inhibiting DNA polymerase II.

Genistein (4′,5,7-trihydroxyisoflavone) is the most important isoflavonoid known, and it is abundantly found in Leguminosae (Fabaceae) family (Dixon and Ferreira, 2002). The substance is known to inhibit cancer recurrence and resistance (Fan et al., 2013). Additionally, it slows the growth of tumors that need estrogen (Zhang et al., 2015). Genistein is a preventive medication for chemically generated mammary tumors. The EGFR signaling pathway which also causes cell differentiation is inactivated by Genistein (Zhang et al., 2015). It also has anti-oxidation, anti-cancer, anti-proliferation properties and is known to inhibit angiogenesis and metastasis and promotes death Fig. 2 (Li et al., 2013, Latocha et al., 2014). By controlling epigenetic processes, genistein is hypothesized to influence gene transcription (Li et al., 2013). Moreover, it inhibits aberrantly activated DNA polymerase II, topoisomerase I and II, and several proteins implicated in cell cycle such as Wee1, cyclin D1, cyclin B1 and CDK-1. The apoptosis inhibitors survivin, XIAP, Bcl-2 and IAP and are all targeted by genistein. Additionally, it boosts the upregulation of several important proteins that are important in regulating cell cycle proliferation like p53, p21, p27, and p16 in cancer cells. Furthermore, tyrosine kinases the important components of signaling networks that control cell viability and proliferation are also suppressed by genistein, which prevents cancer cell survival and growth. Additionally, it decreases the proteolysis of cancer-related tissue and prevents angiogenesis via altering the VEGF, PTK, and MAPK genes (Latocha et al., 2014, Sofi et al., 2022b). It could therefore be used as a medicinal agent to treat different malignancies and breast cancer as well.

Once genistein inhibits the Hedgehog-Gli1 signaling pathway, breast cancer cells undergo cell death by downregulating the proteins SMO and/or Gli1, which eventually impede the cancer stem cell survival pathway, it also limits the proliferation of BCSCs and lowers the endurance of cancer stem cells (CSCs). It has been demonstrated that genistein lowers ALDH1 mRNA levels. A decrease in BCSC stemness is associated with downregulation of ALDH1 and the Hedgehog-Gli1 signaling pathway. Thus, genistein serves as an effective drug for breast cancer prevention by reducing the generation of these stem cells connected to metastasis, resistance to therapy, and cancer relapse (Fan et al., 2013, Qayoom et al., 2021). CDC20, BUB1, MCM2, and cyclin B1 are among the deregulated cell cycle proteins that are impacted by genistein in breast tumor cells. As a result, the cell cycle progression is detained at numerous points, including the G0/G1, G2/M, and G1 phases (Zhang et al., 2015). Genistein also restores the activator-dependent cellular responses to 17-estradiol. Consequently, it is a viable treatment for breast cancer since it targets ER reactivation (Li et al., 2013). Apoptosis is triggered by genistein in T47D cell lines in which there rise in ER-β and cytochrome *c* oxidase levels which leads to a decline in the ratio of ATP synthase to cytochrome *c* oxidase (Pons et al., 2014). By strengthening the inhibition of p-Akt and IGF-1R, genistein reduces the development and causes the death of breast cancer cells. Additionally, genistein lowers the Bcl-2/Bax ratio, indicating that it may be able to stop the progression of breast tumor cells (Liu et al., 2016). This drug may improve the prognosis for individuals with breast cancer while they get therapy (Latocha et al., 2014, Singh et al., 2017).

## 4 Withaferin A

**Fig. 3:**
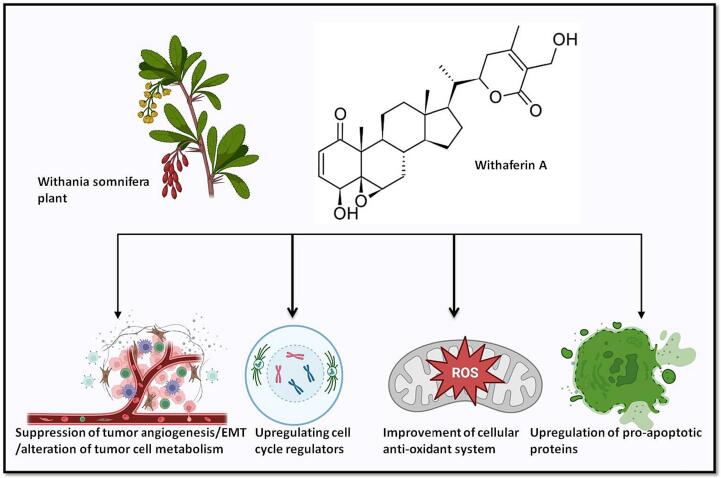
Diagram showing the routes that Withaferin A targets. By blocking the epithelial-mesenchymal transition, selectively inhibiting tumour cell proliferation, and inducing apoptosis by upregulating proapoptotic proteins like Bak, Bax, and Bim and downregulating antiapoptotic Bcl-2 and apoptosis inhibitory proteins like XIAP and c1IAP that survive in breast cancer, withaferin A suppresses tumours. via increasing p53 and p21 to cause cell cycle arrest. Finally, but just as importantly, it strengthens the antioxidant and/or detoxifying systems within cells.

The steroidal lactone Withaferin A is obtained mostly from the plant *Withania somnifera*, often known as the Indian winter cherry. This plant's historical usage in Ayurvedic medicine for curing ailments including inflammation, cancer, and neurological disorders is significant (Bhattacharya et al., 1997, Mohan et al., 2004, Vyas and Singh, 2014). Research in both *in vivo* and in vitro*,* have shown that withaferin A suppresses tumor metastases that is created in an experimental setting. The main factor contributing to its therapeutic effectiveness is its potent pro-apoptotic, anti-proliferative, and antioxidant characteristics. According to studies, Withaferin A makes cancer cells more susceptible to the currently available chemotherapeutic drugs (Lee and Choi, 2016). Removing cells from oxidative and/or inflammatory stress helps prevent the development of cancer because these stresses affect cellular macromolecules like proteins, DNA, RNA and cause cellular biochemical processes to fail (Hanahan and Weinberg, 2011, Qayoom et al., 2023d). Stromal cells, unique host-derived cells, and inflammatory immune cells all work together to generate tumors, which leads to the creation of a microenvironment inside the expanding tumor mass. The primary cause of many cancer symptoms is intra-tumoral inflammation (Kundu and Surh, 2012). Withaferin A's anticancer actions are mechanistically based on the interference with tumor-specific metabolic processes. To name a few, they are: (1) enhancement of the cellular antioxidant and/or detoxification processes (Manoharan et al., 2009a, Manoharan et al., 2009b, Panjamurthy et al., 2009); (2) preventing the inflammatory pathways (Min et al., 2011, Lee et al., 2012, SoRelle et al., 2013, Mehraj et al., 2021c); (3) apoptosis induction and selective tumor cell proliferation inhibition; (4) reducing tumor angiogenesis; (5) preventing tumor invasion and metastasis, and epithelial-to-mesenchymal transition; (6) metabolic changes in tumor cells; (7) By raising the cell cycle controllers p53 and p21 as well as the tumor-promoting proteins Bim, Bax, and Bak in tumor cells of the breast and reducing the antiapoptotic protein Bcl-2 and the death protein inhibitors XIAP and c1IAP Fig. 3 (Stan et al., 2008, Zhang et al., 2011, Hahm and Singh, 2013). Other tumors have also been examined for WA's potential anticancer action, including leukaemia, cervical cancer, glioblastoma, melanoma, and pancreatic cancer cells (Malik et al., 2007, Yu et al., 2010, Mayola et al., 2011, Munagala et al., 2011, Grogan et al., 2014). It affects vimentin disassembly and phosphorylation as well as FOXO3a-Bim dependent apoptosis and thereby prevents breast carcinoma progression (Thaiparambil et al., 2011, Qayoom et al., 2023a). It's imperative to note that while these results are promising, the transition from preclinical development to clinical trials involves additional steps and considerations. The safety, efficacy, and optimal dosage of Withaferin A need to be thoroughly evaluated in human subjects. Additionally, regulatory approvals and ethical considerations are critical aspects of progressing a potential therapy through clinical development. If Withaferin A continues to show promise in further studies, it may represent a novel approach or supplementary therapy for breast cancer. As research progresses, it is advisable to stay updated on the latest findings through scientific publications and discussions with healthcare professionals.

## 5 Biochanin A

Biochanin A is indeed an isoflavone, and it is found in various plants, including red clover (Trifolium pratense). Red clover has been a traditional medicinal plant, and it contains several bioactive compounds, with isoflavones being among the key constituents. Research has been done on the possible health advantages of biochanin A, particularly its anticancer characteristics, in conjunction with other isoflavones for instance: genistein and daidzein. The potential anticancer effects of biochanin A are attributed to several mechanisms, and research in this area is ongoing (Heinonen et al., 2004). The compound was suggested to manage the metabolic activation of pro-carcinogens. This is important as the activation of certain chemicals can add to the initiation and succession of carcinoma. Biochanin A demonstrated the ability to reduce mRNA expression and restrain the action of the aromatase enzyme in SK-BR3 cells. SK-BR3 cells are breast carcinoma cells that lack the estrogen receptor (ER). In these cells, the inhibition of aromatase may be relevant for suppressing estrogen-independent mechanisms of cancer growth. The biochanin A metabolite genistein was reported to inhibit the activation of promoter I.3/II. This finding is significant as promoter I.3/II is associated with the regulation of aromatase expression. Inhibiting this promoter can contribute to reducing aromatase activity. Biochanin A was reported to be more tolerable than genistein in studies using MCF 12A and MCF7 cell lines. Furthermore, the chemical was observed to upregulate tumor repressor gene expression. Tumor repressor genes are essential for regulating cell division and postponing the onset of cancer. It's crucial to remember that these results are exclusive to the experimental setups that the study detailed, and more research may be necessary to translate these findings into clinical settings. Given the intricacy of cancer biology and the variability of breast cancer, further research is required to completely comprehend the range of effects of biochanin A and its possibility as a therapeutic agent or prevention approach (Wang et al., 2008, Sehdev et al., 2009, Atwell et al., 2015).

Comparable outcome were reported by Young et al., who found that biochanin A outperformed genistein in terms of tumor suppressor gene expression upregulation (Moon et al., 2007). According to Bhushan et al., biochanin A decreased the survival and signalling of SK-BR-3 carcinoma cells in addition to the synthesis and activity of invasive enzymes (Sehdev et al., 2009). In a different study, biochanin A was discovered to be beneficial in preventing the augmentation of estrogen-receptor positive breast carcinoma in the model of xenograft mice (Moon et al., 2008). To completely comprehend the bioavailability, therapeutic approach and metabolic impact of biochanin A in diverse breast cancer patient types, more study in clinical trial settings is required. The function of biochanin A in various breast cancer growth pathways also needs to be clarified. Since ER-positive tumor cells are used in the greater part of experimental studies, future investigation should concentrate more on the distinct role of biochanin A on hormone receptor negative or TNBC cells as well (Moon et al., 2008).

## 6 Tetrandrine

**Fig. 4:**
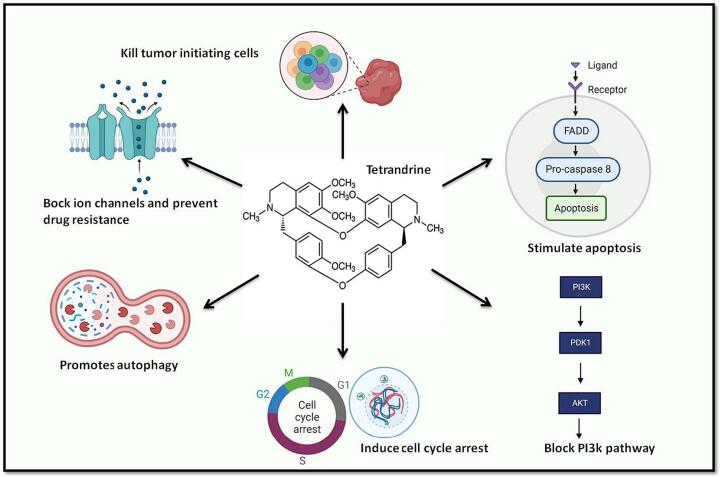
The diagrammatic representation displays the targets of tetrandrine in breast cancer. Tetrandrine is an autophagy activator that accelerates cell death in apoptosis-resistant cells and stimulates autophagy in a variety of BrCa cell lines. Less Bax, Caspase 3, and Caspase 7 are seen in cell types that are resistant to apoptosis. Tetrandrine causes cell cycle arrest, upregulates P53 and CIP/KIP family proteins, and prevents cells from entering the G1 phase. The pharmacologic properties of tetrandrine help to prevent the development of drug resistance proteins and to block positive ion channels. Additionally, tetrandrine inhibits the PI3K/AKT/mTOR pathway, which is required for angiogenesis, cell migration, survival, and proliferation. Tetrandrine kills the inflammatory and breast cancer-causing cells to prevent them from reproducing.

Tetrandrine has anti-proliferative effects and prevents the spread of carcinoma cells (Lan et al., 2018). It is extracted from the Asian herb (Chinese plant) *Stephania tetrandra* (Xu et al., 2011, Lan et al., 2018). Prostate cancers, leukaemia, melanomas, and breast malignancies have all been shown to respond to this natural chemical by induction of apoptosis (Lan et al., 2018). Tetrandrine has pharmacologic effects that assist to inhibit positive ion channels and prevent drug resistance proteins (Xu et al., 2011). This substance modifies tumor cells' resistance (Jiang et al., 2017) and are capable of overcoming drug resistance in human breast carcinoma (Chen and Chen, 2013). Tetrandrine also promotes autophagy in cells that are tolerant of apoptosis (Wong et al., 2017). Tetrandrine is a hopeful medication for the diagnosis of several carcinomas, including breast carcinoma, since it stops cancer cells from reproducing. Tetrandrine stops the multiplication of breast tumor-initiating and inflammatory cells by killing them. This medication decreases aldehyde dehydrogenase (ALDH1) protein expression and mammosphere formation, two indicators of cancer cell proliferation. Tetrandrine has an antiproliferative effect on TNBC breast cancer cells. Aldehyde dehydrogenase proteins also contribute to cell progression in breast cancer cells hence their downregulation induces an anti-proliferative effect (Antonescu Mintas et al., 2021). It has been demonstrated that tetrandrine may eliminate tamoxifen resistance in MCF-7/TAM cells. Tetrandrine efficiently causes cancer cells to die by escalating the activity of Caspase 7, Bax, and Caspase 3, which are produced at lower levels in apoptosis-resistant cell types Fig. 4 (Wong et al., 2017). Thereby making it a promising treatment for breast cancer (Chen and Chen, 2013). By either activating caspase or by using the FASL-mediated apoptotic pathway, Tetrandrine has been shown to have the ability to stop cells from growing, which causes them to undergo apoptosis. Tetrandrine inhibits CDKs' ATP-binding sites and prevents cells from going through the G1-S transition, suppressing CDK2-CycE and CDK4 in colon, endothelial, and liver carcinomas (Kuo and Lin, 2003). Tetrandrine has also been found to have an impact on these proteins at pharmacological dosages, except CDK1, CDK2, and CDK6. Tetrandrine increased P53 and the CIP/KIP family proteins in a time-specific way in a numeral malignancy, preventing the cells from reaching the G1 phase (Kuo and Lin, 2003, Chen et al., 2014). Tetrandrine also decreased hyperphosphorylated “RB” levels, which may serve to inhibit CDK4, CycD1 and CDK6 levels that would otherwise promote the shift from G1 to S (Kuo and Lin, 2003, Chen et al., 2014, Qayoom et al., 2022). It is believed that tetrandrine activates CycD1, or cyclin D1, a protein that combines with CDK to generate a complex that reins the G1 stage of the cell cycle. Additionally, the mention of “proteolysis” indicates that tetrandrine may be involved in the degradation of CycD1. Proteolysis is the process of breaking down proteins, and in the context of the cell cycle, it can be a regulatory mechanism to control the levels of specific proteins. It is claimed that tetrandrine activates a GSK3-regulated pathway. GSK3 is a kinase that regulates the cell cycle among other biological functions. It can influence the steadiness and degradation of proteins, including those involved in the cell cycle. Tetrandrine is compared to other compounds, such as resveratrol, cycloheximide, and aspirin, in terms of its ability to block CDK and activate CycD1. Resveratrol is a natural compound found in certain plants, cycloheximide is an antibiotic, and aspirin is a common non-steroidal anti-inflammatory drug (NSAID). Each of these compounds may have distinct mechanisms of action. It's vital to note down that while these conclusions suggest potential anticancer properties for tetrandrine, the translation of these laboratory observations into clinical applications requires further research and validation. The complexity of cellular signaling pathways and the potential for off-target effects underscore the need for comprehensive studies to comprehend the means of action and potential side effects of tetrandrine. If tetrandrine or related compounds prove effective as CDK inhibitors, they could potentially be explored as therapeutic agents in cancer treatment. However, clinical trials and rigorous testing are necessary to assess their safety, efficacy, and specificity for cancer cells. Always consult with healthcare professionals or researchers for the latest information on the development and potential applications of such compounds in cancer therapy (Mehraj et al., 2022a). Blocking the PI3K/AKT/mTOR pathway is a targeted approach in carcinoma therapy. In cancer cells, this pathway is frequently hyperactivated, leading to uncontrolled cell growth and survival. Tetrandrine could have an impact with the survival and angiogenesis of cancer cells by blocking this route, which makes it a viable option for more research as an anticancer drug. It's important to note that the specific mechanisms through which tetrandrine blocks the PI3K/AKT/mTOR pathway, as well as its potential side effects and therapeutic window, would require further detailed investigation. The preclinical findings in murine endothelial cells provide a basis for further research into tetrandrine's potential as a targeted therapy for diseases involving dysregulated PI3K/AKT/mTOR signaling, including certain cancers. As with any potential therapeutic agent, the transition from preclinical studies to clinical applications requires rigorous testing, including in vitro studies, animal models, and eventually human clinical trials, to establish safety and efficacy. Always consult with healthcare professionals or researchers for the latest information on the development and potential applications of tetrandrine in the context of the PI3K/AKT/mTOR pathway and cancer therapy (Kuo and Lin, 2003, Lee et al., 2010, Chen et al., 2014).

## 7 Garcinol

**Fig. 5:**
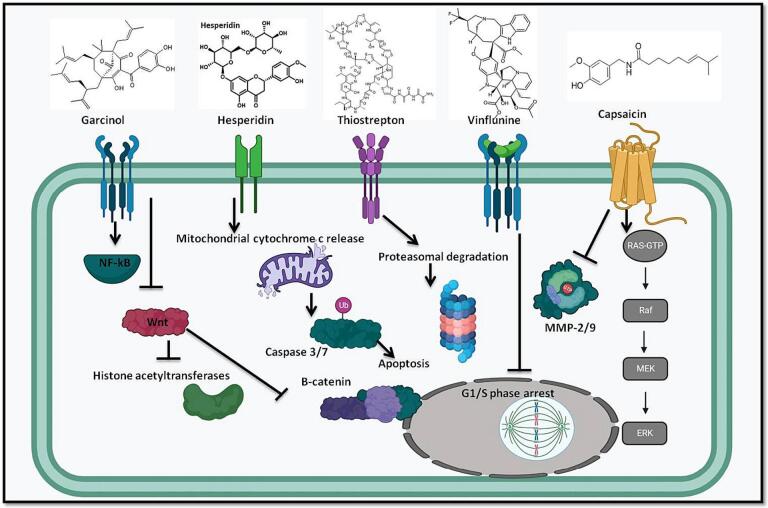
Diagram showing how natural substances that target breast cancer signalling pathways are arranged. Garcinol reduces ROS and inhibits histone acetyltransferases. Garcinol inhibits the production of nuclear catenin, vimentin, miRNAs, and NF-kB, all of which are essential for the growth of tumour cells. By activating caspase-8, hesperidin triggers p53-dependent apoptosis via the mitochondrial-dependent apoptotic pathway. Upon activation, caspase-8 produces cytochrome *c*, which in turn activates caspase-9, which in turn activates caspase-3 and-7, ultimately leading to apoptosis. To target the proteasome and aid in lowering resistance, thiostrepton may directly interact with the FOXM1 protein. Vinflunine selectively binds to tubulin at vinca alkaloid binding sites to prevent microtubule polymerization. The cell cycle is halted in the G2/M phase due to a blockage of the mitotic spindle formation, which results in apoptosis and cell death. In addition to its anticancer properties, capsaicin also lowers the expression of AMP-activated protein kinase, matrix metalloproteinase-9 (MMP-9), and nuclear factor-kappaB (NF-kB).

Garcinol is a polyisoprenylated benzophenone, which means it has a specific structure with multiple isoprene units. Its chemical structure contributes to its biological activity. Garcinol is found in the fruit rind of the Garcinia plant, particularly in Garcinia indica. The fruit is native to regions such as India, Africa, and China. Garcinia plants, including those producing garcinol, are highly valued in traditional medicine and cuisine in India, Africa, and China. In these regions, a range of parts of the plant are used for culinary, medicinal, and cultural rationales. In traditional medicine, garcinol and extracts from Garcinia plants have been used for various health-related purposes. Their anti-inflammatory, antioxidant, and anticancer qualities are frequently ascribed to them. Garcinol's range of biological actions has been investigated. It has been studied for its probable as an anticancer drug and has demonstrated antioxidant qualities. Studies have indicated that garcinol might impact a number of cellular functions, such as the control of the cell cycle, the suppression of certain signaling pathways, and apoptosis (programmed cell death) (Saadat and Gupta, 2012). Garcinol is used more frequently for its ability to treat cancer than for its antioxidant properties. Garcinol inhibits histone acetyltransferases (HDAC) and lowers ROS levels, as a result it seems to be an effective cancer treatment Fig. 5 (Ahmad,et al., 2012). It has been shown that garcinol decreases the quantity of estrogen (E2)-induced synthesis in breast cancer cells MCF-7. It is well recognized that estrogen contributes to the emergence and spread of hormone receptor-positive breast cancer. It is claimed that garcinol inhibits NF-kB/p65′s nuclear translocation. Because NF-kB's nuclear translocation is a crucial step in its activation, which consequences in the transcription of genes concerned in inflammation and cell survival, this function is important. Garcinol has been shown to prevent several genes, such as cyclin D1, Bcl-2, and Bcl-xL, from synthesizing mRNA. Cell cycle regulation is regulated by cyclin D1, whereas anti-apoptotic proteins are Bcl-2 and Bcl-xL. Apoptosis and the course of the cell cycle can be affected by inhibiting their production. According to the data, garcinol alters gene expression to prevent breast carcinoma cells from proliferating. Significant pathways implicated in the initiation and spread of cancer is probably impacted by this alteration in gene expression. It is said that garcinol targets the NF-kB pathway, a signaling mechanism linked to both cancer and inflammation. Cancer research and therapy use the technique of inhibiting the NF-kB pathway to regulate inflammation and cell viability. These findings collectively suggest that garcinol may have potential as a therapeutic agent for breast cancer, particularly in MCF-7 cells. However, it's important to note that the translation of these laboratory observations into clinical applications requires further research, including detailed preclinical studies and eventually clinical trials, to establish the safety and efficacy of garcinol in human subjects (Ye et al., 2014). By obstructing the Wnt signaling pathway, garcinol keeps cancer cells from invading healthy tissue. Additionally, it has been shown that in mouse mammary glands, garcinol decreases the amounts of vimentin, nuclear β-catenin, miRNAs, and NF-kB (Park et al., 2009). Additionally, by limiting cell migration through a decrease in 9-nAChR and cyclin D3 levels, this chemical trigger apoptosis and limits the expansion of breast carcinoma cells. Research on garcinol and its budding role in breast carcinoma is ongoing, and it's important to note that scientific understanding can evolve. As of my last update, there have been some studies suggesting that garcinol may have anti-cancer properties, including inhibitory effects on various signaling pathways associated with cancer development. Several pathways are implicated in breast cancer, including those involving inflammation, cell progression, and death. Some studies have suggested that garcinol may affect these pathways, potentially inhibiting the augmentation and progression of breast carcinoma cells. To completely comprehend the processes and ascertain if garcinol is helpful in diagnosing or stopping breast carcinoma, additional study is necessary (Vinod et al., 2015) (Ahmad et al., 2010).

## 8 Hesperidin

Hesperidin is a major flavanone glycoside found in citrus fruits (Kanaze et al., 2007). Hesperidin may have anti-cancer effects on breast cancer cells, influencing multiple pathways involved in cell survival, apoptosis, invasion, and stemness. It is supposed to have an impact on the expression of genes associated to apoptosis, such as bcl-2, as well as EMT indicators like MMP-9, stemness genes like ALDH1 and cycle proteins like P21, and cyclin D1. Hesperidin was shown to dramatically upregulate the P53 and cyclin D1 mRNA levels while downregulating the Bcl-2, ALDH1, and MMP-9 mRNA levels (Yap et al., 2021). Hesperidin is also revealed to repress the NF-kB and Akt pathways that eventually leads to decreased PD-L1 expression levels in breast cancer (Kongtawelert et al., 2020). The ATP-binding site in the tyrosine kinase domain exhibits considerable contact with it, which limits ATP binding. It was also found to have inhibitory effects on tyrosine kinase activity in breast carcinoma (Chandrika et al., 2016). Hesperidin's actions on possible breast cancer targets suggest the need to investigate this substance's anti-cancer treatment potential for diagnosis.

## 9 Thiostrepton

The natural peptide macrocycle thiostrepton, which is made up of heterocyclic rings with sulphur, belongs to the thiopeptide class of peptides. an organic substance well-known for its strength as an antibiotic (Bagley et al., 2005). Thiostrepton may effectively associate with FOXM1 protein. This suggests that thiostrepton and FOXM1, a crucial regulator of cell cycle progression, could interact substantially. By binding to FOXM1, thiostrepton may impact the activity or stability of FOXM1. Targeting the proteasome: The information suggests that thiostrepton's interaction with FOXM1 may involve targeting the proteasome. Proteasomal degradation is a common mechanism for controlling the levels of regulatory proteins, and it appears that thiostrepton may influence FOXM1 degradation. It's interesting to note that, in contrast to stabilizing P53 and P21, the reported mechanism of action for thiostrepton in this context is associated with its interaction with FOXM1 and its impact on proteasomal activity. These results imply that thiostrepton may have a therapeutic effect in breast cancer via influencing cell cycle regulation and FOXM1 targeting. But like with any research findings, more investigation is compulsory to corroborate these results and evaluate the efficacy and effectiveness of thiostrepton as a possible breast cancer therapy, including preclinical and clinical trials Fig. 5 (Bhat et al., 2009). Thiostrepton, according to Watanabe et al., may also adhere to a mitochondrial ribosomal component and impair the mitochondrial translational mechanism (Zhang et al., 2005). Thiostrepton, according to Hegde et al. (2011), binds to the FOXM1 protein directly and prevents it from interacting with several gene promoters and that this transcriptional factor does not bind to its own promoter is peculiar. However, despite sharing the DNA-binding domain with FOXM1, only a small number of other fork head family members (FOXA1, FOXO1, FOXO3a, and FOXO) demonstrated resistance to thiostrepton's suppressive effects. These results support the idea that the FOXM1 thiostrepton binding site should be located in a specific location and function as an allosteric regulator (Hegde et al., 2011). Additionally, it has been shown that Thiostrepton, which targets the Sonic Hedgehog (SHH) pathway, inhibits the proliferation of the CD44+/CD24- stem cell population in TNBC (Yang et al., 2016). We while studying about this compound found that its main target was FOXM1 while another study suggested its role in reducing the cancer stemness via sonic hedgehog pathway; therefore, we speculate that there might be more potential targets of Thiostrepton that needs to be explored further.

## 10 Vinflunine

New fluorinated vinca alkaloid called vinflunine has recently been discovered as a microtubule inhibitor with enhanced anticancer activity and less neurotoxicity (Braguer et al.). Vinflunine was selected to diagnose a variety of solid carcinomas, including breast carcinoma (Bennouna et al., 2003). In a 2007 research, vinflunine and trastuzumab (an anti-HER2 antibody) were found to be a safe and efficient treatment for breast cancer cells with overexpressed HER2 genes (Yardley et al., 2010). Similar to other vinca alkaloids, vinflunine works through inhibiting microtubule polymerization by specifically attaching to tubulin at the binding sites that results in the obstruction of the formation of the mitotic spindle causes the G2/M phase of the cell cycle to be arrested, which then triggers apoptosis and cell death Fig. 5 (Kruczynski et al., 1998). However, compared to other vinca alkaloids, vinflunine binds to tubulin with a weaker affinity that results in less neurotoxicity (Kruczynski and Hill, 2001). Vinflunine has been proven in several *in-vitro* studies to have anti-angiogenic and anti-metastatic properties by reducing the development of vascular endothelial cells (Kruczynski et al., 2006). In an open-label randomized phase III trial (2018) Vinflunine was demonstrated to have a significantly higher disease control rate in metastatic breast cancer as compared to the standard drug with a good safety profile at higher concentration (Cortes et al., 2018). There is interest in vinflunine's further clinical investigation since some experimental models imply that it has a stronger antitumor impact than other related alkaloids (Bennouna et al., 2008).

## 11 Conclusion

Breast cancer is the most frequent type of cancer in women and one of the major causes of death worldwide for people. Several factors are responsible for the progression of breast cancer in females. There have been several reports of disruptions in different biochemical pathways and the corresponding changes in the expression of multiple molecular markers. Even while there are many powerful advantages to treatment approaches including radiation, chemotherapy, and surgery, the negative side effects of these approaches have led researchers to look for alternatives. One such prospective treatment approach is use of phytotherapeutics to target different molecular markers changed during cancer and, as a result, defend against cancer without having any negative side effects. These substances work against multiple signalling pathways, primarily those involved in cell death (apoptosis and autophagy) and embryonic development (Notch pathway, Wnt pathway, and Hedgehog pathway). Thus, we can conclude that phytotherapeutics block the several signalling pathways that act as catalysts for breast cancer development thereby opening the door for the creation and discovery of anticancer medications.

## 12 Future perspective

Breast cancer is among the most prominent cancer in women worldwide and is most common in individuals who are 44–55 years old (Coleman et al., 2008). TNBC differs from other forms of cancer in that it lacks the activation of the HER2, progesterone, and estrogen receptors. 15–20 % of all cases of breast cancer are caused by it. TNBC is typically diagnosed with conventional methods such surgery, chemotherapy and radiation therapy. Chemotherapy given before to surgery is associated with a favorable pathologic response and a raised overall survival, according to earlier research (Sofi et al., 2023). By looking at a cancer cell from tumor tissue that had been exposed during surgery but was not active, the efficacy of preoperative therapy was assessed. Although widely seen as a big benefit, it can also increase resistance. Innovative breast cancer medicines, such as cancer and metastasis-preventing medications, must be developed since TNBC patients who get untargeted therapy portray weak prognosis (Gupta et al., 2020, Qayoom et al., 2023c). The majority of research focuses on cancer treatments made from natural materials, especially phytochemicals. Molecular targets such as genes can be affected directly by natural molecules known as phytochemicals, or they can indirectly affect metabolic processes via stabilising conjugates. Due to their capability to inhibit EMT, increase apoptosis, and promote invasion, phytochemicals may be a viable molecular targeted treatment to inhibit several signaling pathways involved in cancer metastasis for instance, NF-kB, Wnt/β-Catenin, PI3K/Akt/mTOR, RAF/MEK/ERK, STAT-3, EGFR and Hedgehog (Dewi et al., 2022, Qayoom et al., 2023b). Today, natural chemicals are recognized as anti-cancer agents because of their safety, lack of negative side effects, and capacity to lessen chemotherapy treatment resistance. Additionally, by concentrating on several cancer signaling pathways in malignancies like triple negative breast carcinoma, they increase the efficacy and antiproliferative effects. This study focuses on many naturally occurring bioactive compounds produced from plants that may have an inhibitory effect on related signaling cascades to prevent breast cancer.

## 13 Authors’ contributions

AA designed and supervised the work. AA wrote the manuscript, designed the figures, critically revised and edited the manuscript.

## Funding

The funding was provided by Deanship of Scientific Research University of Al Baha Saudi Arabia via Grant No. R-2023-702 to Abdullah Almilaibary.

## Declaration of competing interest

The authors declare that they have no known competing financial interests or personal relationships that could have appeared to influence the work reported in this paper.
